# Corrosion Performance of Nano-ZrO_2_ Modified Coatings in Hot Mixed Acid Solutions

**DOI:** 10.3390/ma11060934

**Published:** 2018-06-01

**Authors:** Wenhua Xu, Zhenyu Wang, En-Hou Han, Shuai Wang, Qian Liu

**Affiliations:** 1 Key Laboratory of Nuclear Materials and Safety Assessment, Institute of Metal Research, Chinese Academy of Sciences, Shenyang 110016, China; whxu13b@imr.ac.cn (W.X.); ehhan@imr.ac.cn (E.-H.H.); shuaiwang@imr.ac.cn (S.W.); qliu17s@imr.ac.cn (Q.L.); 2 University of Chinese Academy of Sciences, Beijing 100049, China; 3 School of Materials Science and Engineering, University of Science and Technology of China, Hefei 230026, China

**Keywords:** nano coatings, EIS, corrosion performance, acid corrosion

## Abstract

A nano-ZrO_2_ modified coating system was prepared by incorporation of nano-ZrO_2_ concentrates into phenolic-epoxy resin. The corrosion performance of the coatings was evaluated in hot mixed acid solution, using electrochemical methods combined with surface characterization, and the effects of nano-ZrO_2_ content were specially focused on. The results showed that 1% and 3% nano-ZrO_2_ addition enhanced the corrosion resistance of the coatings, while 5% nano-ZrO_2_ addition declined it. The coating with 3% nano-ZrO_2_ presented the minimum amount of species diffusion, the lowest average roughness (5.94 nm), and the highest C/O ratio (4.55) and coating resistance, and it demonstrated the best corrosion performance among the coating specimens.

## 1. Introduction

The flue gas from oxy-fuels has higher levels of acid gases, mainly including SOx and NOx with smaller amounts of HF and HCl [1]. The hot flue gases may be compressed and cooled to form mixed acid environments, which are mainly composed of highly concentrated H_2_SO_4_, HNO_3_, HCl and HF. With pH values that are usually in the range of 0–2, the mixed acids environment promotes an extremely high rate of steel corrosion, threatening the safety and operation of equipment.

Organic coatings have been used to control the corrosion of steels in the highly acidic environment. Phenolic-epoxy resin is the most important and industrialized epoxy polymer and possesses excellent acid and alkali resistance [2,3]. It acts as a physical barrier layer between the metal substrate and solution to inhibit the permeation of water and aggressive ions [4]. However, aggressive ions can easily attack coatings through the pores, especially strong acid ions. Due to the presence of porosity [5] and inherent brittleness [6], many coatings used in these conditions are too intolerant for strong acid at high temperature, which leads to premature failure [7].

In recent years, nanoparticles in the coating systems have shown outstanding properties, i.e., excellent barrier resistance, flame retardancy, and wear resistance, etc. [8,9,10,11,12], which have attracted more and more attention. The barrier properties of the organic coatings can be improved by the inclusion of proper fillers, and nano-sized fillers have superior barrier properties than conventional fillers, even at low concentrations, due to their higher surface area [13]. Among these nanoparticles, ZrO_2_ nanoparticles are one of the most promising types of pigment [14] because of its advantages, such as remarkable chemical stability, wear resistance, strength and fracture toughness, and chemical resistance to extreme environments [15,16]. However, very few studies of organic coatings modified by nano-ZrO_2_ in hot mixed acid have been reported.

In the present work, nano-ZrO_2_-modified coatings are formulated to protect steel components in the acid industrial environment. The open circuit potential method (OCP), electrochemical impedance spectra (EIS), X-ray photoelectron spectroscopy (XPS), scanning electron microscopy (SEM), transmission electron microscopy (TEM), atomic force microscopy (AFM) and Raman spectra are utilized to characterize the effect of nano-ZrO_2_ content on the corrosion resistance of composite coatings in mixed acid solution (including 3 wt % H_2_SO_4_, 1 wt % HCl, 0.5 wt % HNO_3_, and 0.2 wt % HF) at 60 °C, which simulates the acidic environment in petrochemical plants. An anticorrosion mechanism of composite coatings is also proposed.

## 2. Materials and Methods

### 2.1. Sample Preparation

Phenolic-epoxy resin (epoxide equivalent per weight: 172–179 g/eq, viscosity (51.7 °C): 1100–1700 mPa·s, and density (25 °C): 1.21 g/mL, Sinopharm Chemical Reagent Co. Ltd., Shanghai, China), phenolic modified aromatic amine (HN01) (amine value: 290 ± 10 KOH mg/g, viscosity (25 °C): 960 mPa·s, and activated hydrogen equivalent per weight: 86, Sinopharm Chemical Reagent Co. Ltd., Shanghai, China) and ZrO_2_ (type: HTZr-02, purity ≥ 90, grain size: 40 nm, surface area ≥ 20 m^2^/g, and crystal form: tetragonal phase, Xiaohuang Nano Technology Co. Ltd., Shanghai, China) were used in this study. Propylene glycol methyl ether acetate (PMA), BYK106 dispersant, KH550 silane coupling agent, 240 mesh glass flake and other chemicals were from Shanghai Lingfeng Chemical Corp., Shanghai, China.

### 2.2. Preparation of Nano-ZrO_2_ Concentrates

Nano-ZrO_2_ particles were dried in a vacuum at 120 °C for 72 h before use. Twenty-three percent PMA and 10% BYK106 dispersant were charged into a flask. The mixture was dispersed for 10 min by high speed stirring, and then 67% nano-ZrO_2_ powders were slowly added into it. The new mixture was kept on high speed stirring for another 30 min, and centrifuged for 2 min. Finally, the mixture was ground for 3 h in a ball mill to obtain nano-ZrO_2_ concentrates.

### 2.3. Preparation of Nano-ZrO_2_-Modified Coatings

Sixty-five percent phenolic-epoxy resin, 0.5% KH550 silane coupling agent, 34.1% 240 mesh flake, and 0.4% BYK106 dispersant were mixed and ground for 8 h in a ball mill to obtain the Z-0 coating. Furthermore, 1.49%, 4.48%, and 7.46% nano-ZrO_2_ concentrates (namely 1%, 3% and 5% nano-ZrO_2_) were incorporated into the Z-0 coating and the nano-ZrO_2_-modified coatings, Z-1, Z-3 and Z-5, were produced. Four coatings were spread to one side of the 50 mm × 50 mm Q235 steel plates, and the coated plates were dried at room temperature for 48 h and then 50 °C for 5 h. The coating thickness was 80 ± 5 µm and was used for the EIS test. The composition of the coatings is listed in Table 1.

### 2.4. Particle Size Analysis of Nano-ZrO_2_ Concentrate

Particle sizes were measured using the dynamic light scattering (DLS) technique with the Malvern Zetasizer (Nano ZS). Before the experiment, a small amount of nano-ZrO_2_ concentrate was dropped into solvent PMA and ultrasonic shaken for 20 min to produce a concentration of 0.5 × 10^−6^. The measurement of particle sizes was performed at room temperature with three repetitions.

### 2.5. Surface Properties

The surface and cross section panels were cut out of the coated plates before and after the static immersion test. In the case of cross-section, it was embedded in bakelite using an XQ-2B mounting press in order to grind with SiC paper up to 2000 grit, and then it was polished. The surface and cross section morphologies of samples were analyzed by scanning electron microscopy (SEM, XL30-FEG-ESEM, Philips, Amsterdam, Holland) at 20 kV equipped with an energy dispersive spectrometer (EDS) to evaluate element distribution, and a gold film was sprayed atop the surface of samples to make it electrically conductive. The surface morphologies of coatings were characterized by atomic force microscopy (PicoSPM II AFM), by Molecular Imaging Corp. (Bruker Corporation, Saarbrucken, Germany), with a scanning area of 5 µm × 5 µm.

X-ray photoelectron spectroscopy (XPS, Thermo, Waltham, MA, USA) analysis was performed with an ESCALAB250 spectrometer to study the carbon-to-oxygen (C/O) variation in polymers before and after acid immersion, using AlKα excitation radiation (hv = 1486.6 eV). The XPS plot size was 500 µm, and the constant pass energy was 50.0 eV. The XPS depth profile was carried out using Ar^+^ ions. The sputtering rate and area were 0.2 nm/s and 2 × 2 mm, respectively. At the same time, the atomic percentages of elements were calculated automatically with a testing instrument. The data was fitted with XPSPEAK4.1. In addition, coatings before immersion were almost the same, and only the Z-0 coating needed to be tested.

### 2.6. Electrochemical Studies

The electrochemical properties of the coatings were investigated using an electrochemical workstation (PAR273A, Princeton Instruments, Princeton, NJ, USA). Measurements were carried out in a three-electrode cell with mixed acid solution (including 3 wt % H_2_SO_4_, 1 wt % HCl, 0.5 wt % HNO_3_, and 0.2 wt % HF) as an electrolyte. The three-electrode cell included a saturated calomel reference electrode (SCE) filled with saturated KCl solution which served as a reference electrode (RE), a platinum auxiliary electrode with an exposure surface of 13 mm × 13 mm as a counter electrode (CE), and the sample with an exposure surface of 12.56 cm^2^ as working electrode (WC). Prior to the electrochemical measurements, the panels were kept in the solution for 30 min in order to stabilize the free corrosion potential.

The electrochemical impedance spectroscopy (EIS) measurements were performed at open circuit potential in an applied frequency range from 100 to 10 mHz, and a sinusoidal perturbation signal with 20 mV amplitude was used. The obtained data were interpreted on the basis of equivalent electrical analogs using ZsimpWin to obtain the fitting parameters.

## 3. Results and Discussion

### 3.1. Characterization of Nano-ZrO_2_ Concentrates

**Figure 1:**
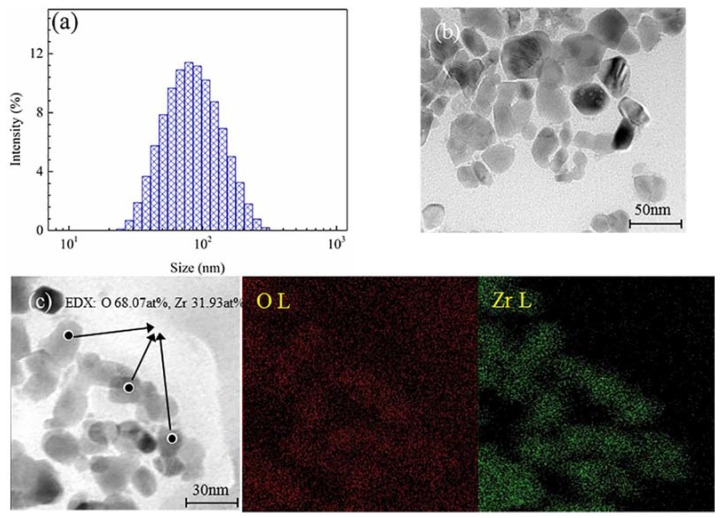
Size distribution (**a**), TEM micrograph (**b**), and chemical element mapping (**c**) of nano-ZrO_2_ concentrates.

The size distribution, TEM micrographs, and chemical element mapping of nano-ZrO_2_ concentrates are shown in Figure 1. The content of particles less than 100 nm in diameter reached 63.09%, with an average diameter of 74.5 nm (Figure 1a). The TEM observations showed that nano-ZrO_2_ particles had a good dispersion state with dimensions of 10–50 nm, and the nano-ZrO_2_ powder appeared to be gray and flat (Figure 1b). The EDS analysis and chemical element mapping showed the presence of elements like oxygen (68.07 at %), and Zirconium (31.93 at %) (Figure 1c), which provides direct evidence for the existence of nano-ZrO_2_.

### 3.2. Acid Immersion Test and Corrosion Morphologies

**Figure 2:**
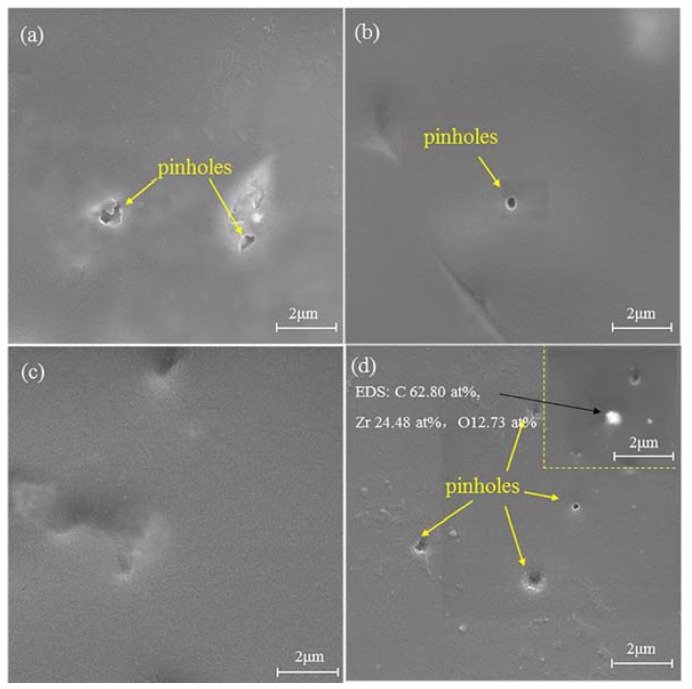
SEM images of different nano-ZrO_2_-modified coatings before acid immersion: Z-0 coating (**a**), Z-1 coating (**b**), Z-3 coating (**c**), and Z-5 coating (**d**).

Typical SEM micrographs for different nano-ZrO_2_-modified coatings before immersion are displayed in Figure 2, along with the EDS analysis in coating Z-5. Pinholes were observed on the surface of the Z-0, Z-1, and Z-5 coatings (Figure 2a,b,d), except for the Z-3 coating (Figure 2c). The aggregation of nano-ZrO_2_ appeared on the surface of the Z-5 coating, which implies that the amount of aggregates increased with the nanoparticle content [17]. This was confirmed by the EDS analysis (Figure 2e), which showed the presence of elements like carbon (62.80 at %), zirconium (24.48 at %) and oxygen (12.73 at %).

**Figure 3:**
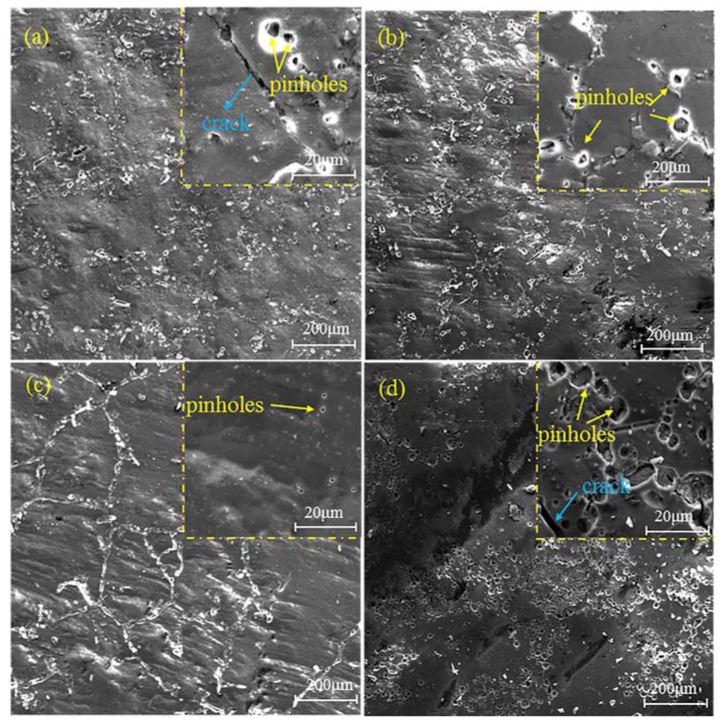
SEM images of different nano-ZrO_2_-modified coatings after 192 h of acid immersion: Z-0 coating (**a**), Z-1 coating (**b**), Z-3 coating (**c**), and Z-5 coating (**d**).

SEM images of different nano-ZrO_2_-modified coatings after 192 h of acid immersion are shown in Figure 3. Various quantities and volumes of pinholes and cracks emerged on the surface of the coatings, which verifies that various levels of degradation occur. Pinholes uniformly distributed on the surface of the Z-0 and Z-1 coatings (Figure 3a,b). Furthermore, pinholes propagated to form cracks on the Z-0 coating. Since 1% nano-ZrO_2_ helps to inhibit the initiation and propagation of cracks, no obvious cracks appeared on the Z-1 coating, which demonstrates that the surface of coating Z-1 has better barrier properties than the Z-0 coating. It is necessary to emphasize that the degradation of the Z-3 coating mainly occurred on particular areas of existing fillers, whilst small pinholes formed, and their quantity and volume were much smaller than other coatings at high magnification (Figure 3c). Three percent nano-ZrO_2_ dispersed uniformly in the Z-3 coating and generated a network nanostructure, which may be responsible for the improvement in corrosion resistance of the Z-3 coating. By contrast, the deterioration of Z-5 coating was most apparent. Non-uniform corrosion and the largest quantity and volume of pinholes were shown (Figure 3d), arising from the aggregation of excessive nanoparticles. Therefore, the surface of the Z-3 coating has the best barrier properties.

**Figure 4:**
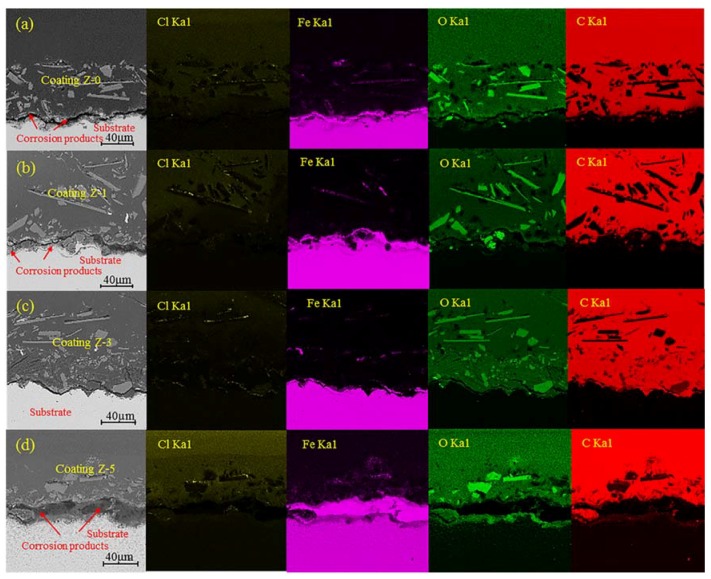
The cross section morphology and chemical element mapping of different nano-ZrO_2_-modified coatings after 192 h of acid immersion: Z-0 coating (**a**), Z-1 coating (**b**), Z-3 coating (**c**), and Z-5 coating (**d**).

Figure 4 exhibits the cross section morphology and elemental mapping for different nano-ZrO_2_-modified coatings after 192 h of acid immersion. The cross section morphology showed that obvious cracks appeared at the interface between the substrate and coatings for the Z-0, Z-1 and Z-5 coatings, especially for the Z-5 coating, while the Z-3 coating remained unchanged.

The elemental mapping revealed that C and O are the major constituents of coatings, and minor amounts of Cl and Fe are also found, which may be introduced from substrates and electrolytes. The big white patches in the oxygen map are glass flakes, and consist of SiO_2_, CaO, and Na_2_CO_3_, corresponding to the low carbon content in carbon map. After 192 h of immersion, corrosive medium went through the coating and reached the metal/coating interface, resulting in the corrosion of substrates. Many iron oxides and hydroxides were generated above the metal substrate, whilst iron ion diffused into the coating via diffusion paths. The concentration of O tended to increase slightly at the metal/coating interface during the immersion period, with the following order: Z-5 coating > Z-0 coating > Z-1 coating > Z-3 coating, corresponding to the distribution sequence of Fe in the coatings. Based on the elemental distributions of O and Fe, different contents of rust formed at the metal–coating interface for these coatings, which revealed that 3% nano-ZrO_2_ effectively prevents the permeation of corrosive medium and reduces the generation of rust, whereas 5% nano-ZrO_2_ promotes the degradation of coatings.

The same diffusion property was observed for Cl and C. The behavior of Cl^−^ may be attributed to its penetration through pinholes [18], which implies that the Z-3 coating possesses remarkable Cl^−^ penetration resistance. Due to the phenomenon of C penetration, degradation of the coatings occurred in the mixed acid, which provided diffusion paths for carbonaceous species dissociated from coatings, and caused C to accumulate at the metal/coating interface. The distributions of Cl and C also demonstrated that the coatings had suffered various levels of degradation, in the order of 5%, 0, 1%, and 3% nano-ZrO_2_ contents.

Internal voids inevitably exist in the coatings, owing to the volatilization of dissolvent and other reasons which provide diffusion paths for small corrosive molecules, such as H^+^, Cl^−^, and O_2_. The diffusion of these corrosive molecules promotes the failure of coatings. The presence of H^+^ evidently accelerates the self-degradation rate of coatings [19], and Cl^−^ reaches the interior of coating through these micro-voids, because of its strong penetration ability. Meanwhile, complete coatings cannot block the attack of acid medium, which causes the nucleation of new micro-voids. Micro-voids grow up with the immersion time, and larger corrosive molecules go through and accumulate at the metal–coating interface, such as carbonaceous species. The nano-ZrO_2_ addition changes the size and amount of internal voids, which has an impact on the corrosion resistance of coatings. The specific effect of nano-ZrO_2_ content on the variation of voids for coatings is discussed in the following EIS sections.

**Figure 5:**
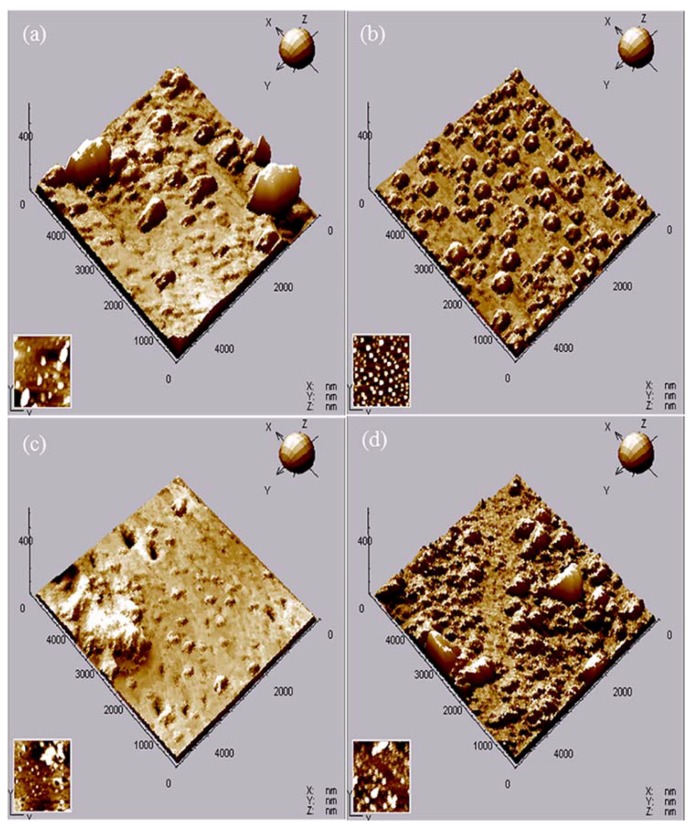
AFM images of different nano-ZrO_2_-modified coatings after 192 h acid immersion: Z-0 coating (**a**), Z-1 coating (**b**), Z-3 coating (**c**), and Z-5 coating (**d**).

AFM images of different nano-ZrO_2_-modified coatings after 192 h immersion are shown in Figure 5, as well as the average roughness (*R*_a_) and root mean square (*RMS*), as deduced from the AFM analysis, are presented in Table 2. Small hill-shaped degradation products were observed on the surface of the Z-0coating, and the main component was carbohydrates. Some flaky degradation products completely separated from the binder due to embrittlement and poor adhesion [20], leading to the highest *R*_a_ (24.58 nm) and *RMS* (73.60 nm) values for the Z-0 coating. With the addition of 1% nano-ZrO_2_, the surface topography for the Z-1 coating changed and refined. Only smaller, regular micro-bulges like hills appeared on the surface, which resulted in a lower *R*_a_ value of 12.79 nm and *RMS* value of 27.44 nm. With an increase in nano-ZrO_2_ content, the smallest micro-bulges were observed on the surface of the Z-3 coating, and the *R*_a_ and *RMS* values reduced to 5.94 nm and 16.10 nm, respectively, in spite of existing amorphous bulges and micro-holes. However, when the nano-ZrO_2_ content reached 5%, the corrosion resistance of the Z-5 coating declined significantly. Patches of micro-bulges became larger, and flaky degradation products were faintly visible, which led to an increase in the *R*_a_ (20.42 nm) and *RMS* values (52.03 nm). In addition, the *R*_a_ value of the Z-5 coating was lower than that of the Z-0 coating, which resulted from the severe inhomogeneous corrosion of the Z-5 coating and a limited scanning area for AFM. In short, the addition of 3% nano-ZrO_2_ minimized the average roughness and root mean square values, which effectively improved the corrosion resistance of the coating.

**Figure 6:**
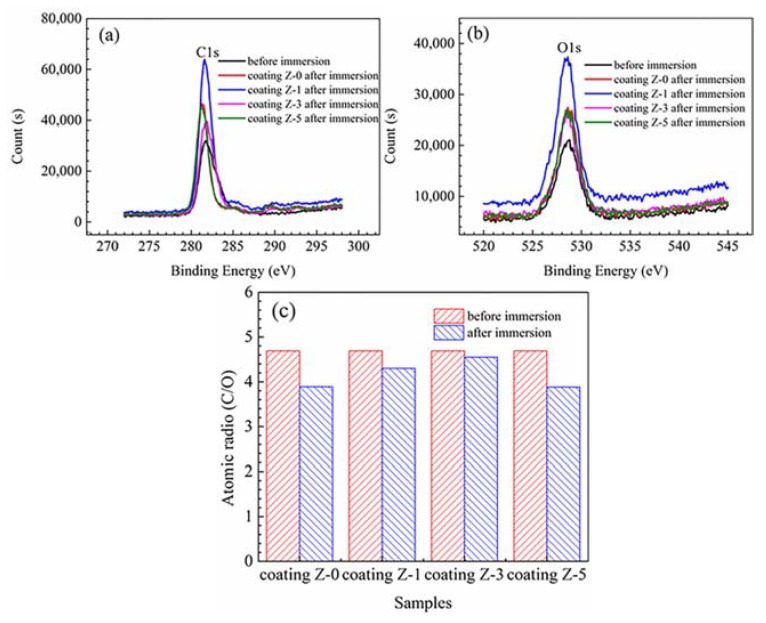
XPS spectra (C1s (**a**), O1s (**b**)) and atomic radio (C/O) (**c**) of the Z-0, Z-1, Z-3, and Z-5 coatings before and after acid immersion.

Due to high surface sensitivity and chemical specificity, XPS was used to better understand the exposure-induced bond failure and component variation in the organic coatings during the corrosion test [7]. Figure 6 shows the XPS spectra (C1s and O1s) and atomic ratio (C/O) of the Z-0, Z-1, Z-3, and Z-5 coatings before and after acid immersion, and the corresponding atomic compositions are given in Table 3. The C1s and O1s XPS peaks of four coatings were at about 281.4 eV and 528.6 eV, respectively. The XPS intensity data were converted into units of at %, and the images provide detailed elemental components. The C1s and O1s spectra were peak-fitted to deduce the concentrations of carbon and oxygen.

Due to strong oxidation and ion permeation at high temperatures, mixed acid initiates the scission of molecular chain and degradation of the polymer network [7]. The general trend is a decrease in C content, which is related to the migration of carbonaceous species from the surface. The amount of O on the surface generally increases, due to the superficial oxidation and water adsorption upon storage [21]. The contents of C, O, and N elements of the Z-0 coating before acid immersion were 79.86%, 17.01%, and 1.80%, respectively, while after acid immersion, these changed to 77.82%, 20.47%, and 1.71%. This is a consequence of the migration of carbonaceous species and superficial oxidation. In addition, the C/O ratio of the Z-0 coating decreased from 4.69 (before acid immersion) to 3.89 (after acid immersion), suggesting that the stability and component of the Z-0 coating was strongly damaged, due to the chemical decomposition.

With the addition of 1% nano-ZrO_2_, the C and O contents after acid immersion for the Z-1 coating increase to 80.37 at % and 18.46 at%, respectively. The C/O value decreases from 4.69 (before acid immersion) to 4.30 (after acid immersion), which is obviously larger than that of the Z-0 coating, demonstrating that 1% nano-ZrO_2_ can inhibit the migration of carbonaceous species and superficial oxidation to a certain degree. With an increase in the nano-ZrO_2_ content, the concentrations of C and O after acid immersion for the Z-3 coating increased to 80.77 at %, and 17.31 at %, respectively. The reduction degree of the C/O value of the Z-3 coating was the smallest, decreasing from 4.69 (before acid immersion) to 4.55 (after acid immersion). The high C/O ratio was directly connected with good anti-oxidation and corrosion resistance, implying that 3% nano-ZrO_2_ provided the physical barrier for corrosive media to permeate, and suppressed the migration of carbonaceous species and superficial oxidation. When the nano-ZrO_2_ content reached 5%, the C content after acid immersion of the Z-5 coating reduced significantly, while the O content increased greatly. Correspondingly, the C/O value after acid immersion (3.88) decreased to the minimum, and it was even lower than that of the Z-0 coating. Excessive nano-ZrO_2_ led to a decline of the anticorrosion property of coatings, and promoted the migration of carbonaceous species and superficial oxidation. In conclusion, XPS analyses showed that Z-3 coating had the best anticorrosion property.

### 3.3. Electrochemically-Evaluated Corrosion Response

**Figure 7:**
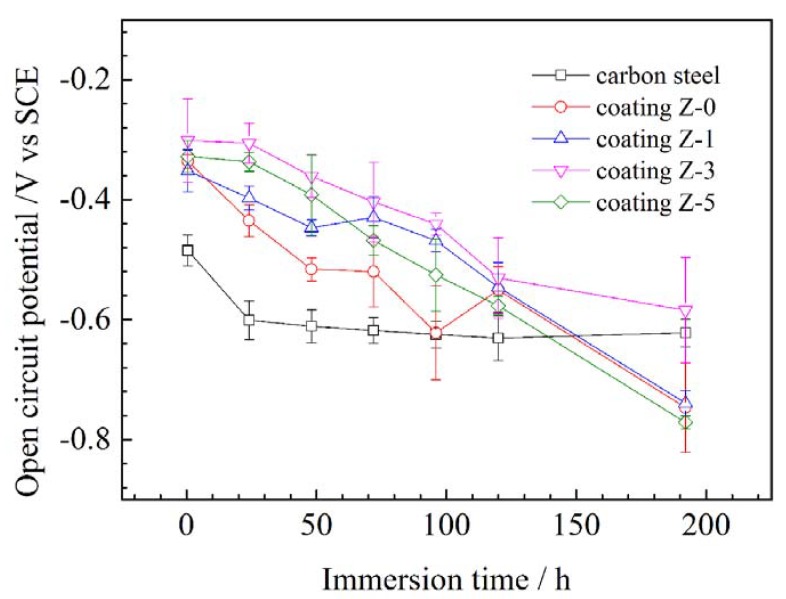
OCP variations of different nano-ZrO_2_-modified coatings in mixed acid solution at 60 °C.

Figure 7 shows the OCP variation in the nano-ZrO_2_-modified coatings with the immersion time. The initial OCP of the bare substrate was –485 mV vs. SCE, and this shifted towards a more cathodic region with time. After 24 h of immersion, it became a steady-state value (–601 mV vs. SCE). In the beginning, the OCP values of the Z-1 coating, the Z-0 coating, the Z-5 coating, and the Z-3 coating were more positive than that of the bare substrate, and increased by 133 mV, 149 mV, 157 mV, and 184 mV, respectively, which clearly indicates the high corrosion resistance provided by these coatings [22]. With a prolonged immersion time, the OCPs of all the coatings presented a similar tendency towards lower values, arising from the diffusion of electrolytes and corrosive ions through coating defects and pinholes [23]. After 120 h of immersion, the potential difference between coatings and bare substrate decreased to 54 mV, 81 mV, 85 mV, and 101 mV for the Z-5 coating, Z-0 coating, Z-1 coating, and Z-3 coating, respectively, indicating that the protection performance of coatings deteriorates continuously with immersion time [24]. After 192 h of immersion, the OCPs of all the coatings reduced towards a negative potential to be lower than bare substrate, except for the Z-3 coating. This is probably due to the heterogeneous reactions arising from the formation of large diffusion paths within coatings [25,26] or the occurrence of some surface phenomena, such as the diffusion of chloride ions through the coating [27]. Consequently, the Z-3 coating exhibited the highest OCP value during the immersion, which demonstrates that the addition of 3% nano-ZrO_2_ effectively improves the anticorrosion property of coatings.

**Figure 8:**
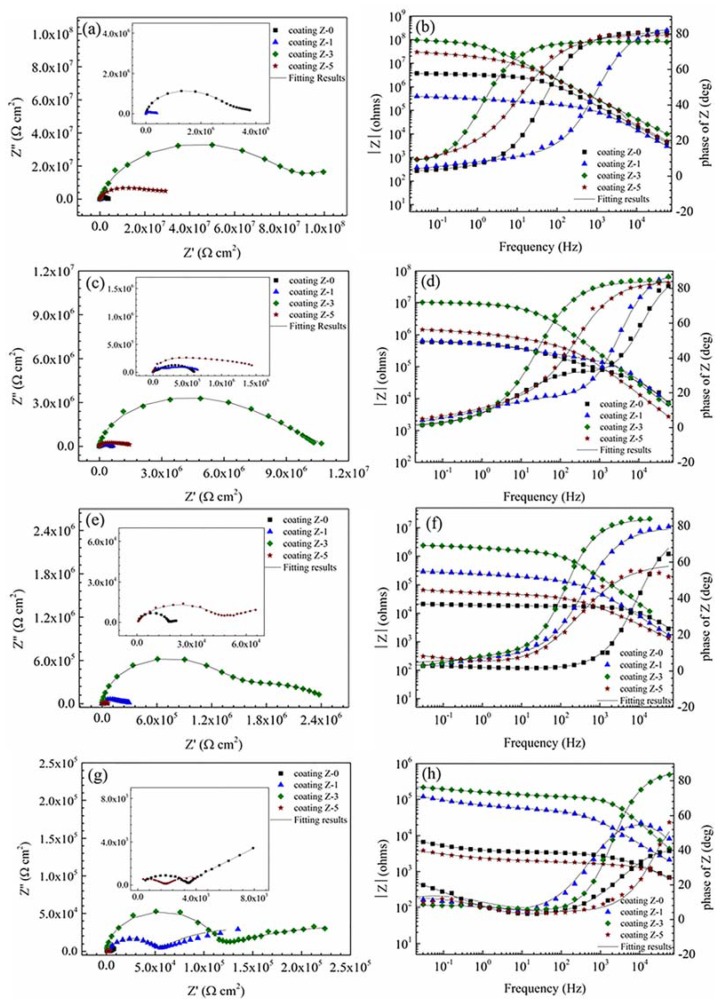
Typical Nyquist and Bode plots for nano-ZrO_2_-modified coatings after different acid immersion times at 60 °C: 30 min (**a**,**b**), 24 h (**c**,**d**), 120 h (**e**,**f**), and 192 h (**g**,**h**).

**Figure 9:**
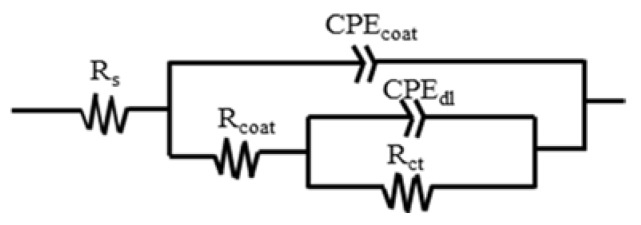
Equivalent circuit model for samples: *R*_s_(*Q*_coat_(*R*_coat_(*Q*_dl_*R*_ct_))).

The typical EIS diagrams measured for various nano-ZrO_2_-modified coatings after 30 min, 24 h, 120 h, and 192 h immersion are presented in Figure 8. The equivalent electrical circuits shown in Figure 9 were used to fit the measured data, and the fitted lines are presented along with the measured data points. The equivalent electrical circuits are addressed in detail in the next section.

The EIS diagrams for all of the coatings showed two time constants. The high frequency time constant shows the barrier properties of the coating, and the low frequency time constant corresponds to the polarization resistance of steel surface beneath the coating layer [23,27,28,29,30]. A shift of phase angle to higher frequency region demonstrates that an increased area of coated substrate is exposed to the corrosive environment [31]. The Z-3 coating showed a high frequency phase angle in the lower frequency region, indicating its superior coating barrier property. The low frequency impedance modulus, |Z|_LF_ (e.g., |Z|_0.01Hz_) is commonly used to roughly estimate the coating resistance [32]. The |Z|_LF_ of the Z-0 coating presented a clear tendency to decrease with the immersion time. After 24 h of immersion, |Z|_LF_ decreased below 10^6^ Ω·cm^2^, and a horizontal line section appeared at the middle frequency, which is characteristic of the delamination of a coating [2,33]. After 192 h of immersion, the |Z|_LF_ was even lower than 10^4^ Ω·cm^2^. The |Z|_LF_ of the Z-1 coating increased firstly and then decreased with the immersion time. Though it was lower in the beginning, the |Z|_LF_ was much higher than that of the Z-0 coating after 192 h of immersion, which testifies that 1% nano-ZrO_2_ has a limited improvement on the anticorrosion property. The |Z|_LF_ of the Z-3 and Z-5 coatings kept decreasing with the immersion time. The |Z|_LF_ of the Z-3 coating was about 10^8^ Ω·cm^2^ in the beginning, and it remained the largest among all the coatings, even after 192 h of immersion, which demonstrates that 3% nano-ZrO_2_ efficiently improves the coating resistance. The |Z|_LF_ of coating Z-5 decreased rapidly from 10^7^ Ω·cm^2^, to a value lower than that of coating Z-0 after 192 h of immersion, which means that an excessive concentration of nano-ZrO_2_ has a negative effect on the anticorrosion property of a coating. Therefore, when the nano-ZrO_2_ content reached 3%, the anticorrosion property of coatings performed best, and then decreased with an increase in the nano-ZrO_2_ content.

The EIS data were fitted by the equivalent circuit *R*_s_(*Q*_coat_(*R*_coat_(*Q*_dl_*R*_ct_))) shown in Figure 9 using ZsimpWin [34]. In these circuits, *R*_s_ is the solution resistance, *Q*_coat_ is the constant phase element (CPE) of a coating, *R*_coat_ is the resistance of the electrolyte in the coating pores, *Q*_dl_ is the CPE of the electrical double layer, and *R*_ct_ is the charge transfer resistance.

The CPE was substituted for pure capacitance, due to surface heterogeneities, deviation from capacitive behavior, and dispersion effects [35]. The impedance of CPE can be written as:

(1) $$Z_{CPE}=\frac{1}{Q{\left(j\omega \right)}^{\alpha }},$$

where *Q* is the CPE constant, *j*$=\sqrt{-1}$, *ω* is the angular frequency (rad/s), and *α* is a CPE exponent associated with the surface heterogeneity or roughness [36]. When *α* < 1, the CPE parameter (*Q*) cannot represent the capacitance, and the effective capacitance (*Ceff*) associated with the CPE can therefore be expressed as [37]:

(2) $$\mathrm{Ce}ff_{\mathrm{coat}}=Q^{\frac{1}{\alpha }}{\left(\frac{R_{s}\cdot R_{coat}}{R_{s}+R_{coat}}\right)}^{\frac{1-\alpha }{\alpha }}.$$

Table 4 presents the electrical element parameters obtained from fitting the measured EIS data in Figure 8. The change in electrical element parameters reflects the change in the electrochemical properties of the coated system [38]. It is generally accepted that the increase in *Ceff*_coat_ with time is related to the water uptake [39] and the dielectric constant [40] of the coating; *R*_coat_ is attributed to the electrical resistance to ionic transfer through the coating pores, which reflects the porosity of coatings and the anti-penetrating ability to the electrolyte solution [31,41]; *R*_ct_ is used to specify the delamination of the top coat and the onset of substrate corrosion [42], which is inversely proportional to the surface area of the sample [43]. In general, a good coating system is characterized by high resistances (*R*_coat_ and *R*_ct_), lower capacitances (*Ceff*_coat_) [44].

**Figure 10:**
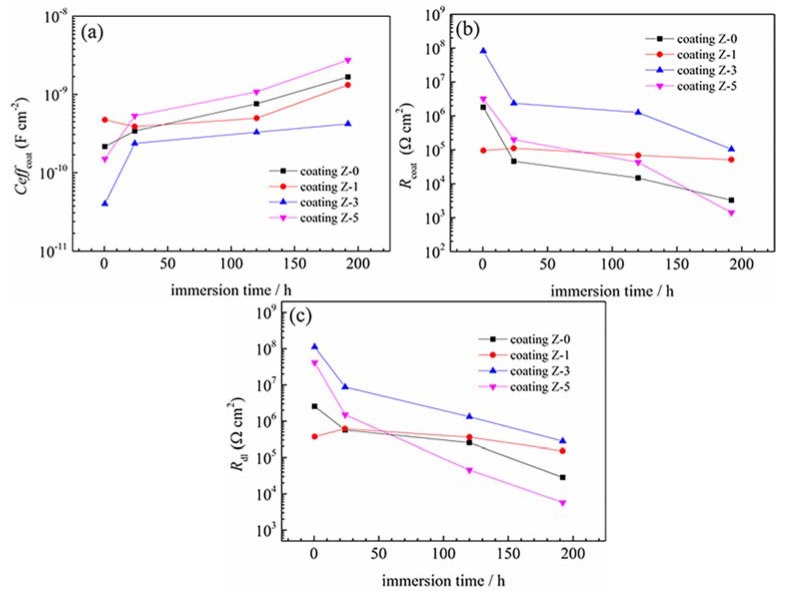
Variations of *Ceff*_coat_, *R*_coat_, and *R*_ct_ with immersion time (values from Table 4).

The plots in Figure 10a–c clearly show the variation trends of *Ceff*_coat_, *R*_coat_ and *R*_ct_ for the coatings versus the immersion time. The *Ceff*_coat_ of all of the coatings was very low (less than 10^−8^), indicating the insulating nature of the coatings [28]. At the beginning of the test, all of the coatings showed high resistances (*R*_coat_ and *R*_ct_) and low *Ceff*_coat_, except for the Z-1 coating. The *Ceff*_coat_ value of the coatings increased in the following order: Z-3 coating, Z-5 coating, Z-0 coating, and Z-1 coating. This shows that the water resistance of coatings does not increase with the nano-ZrO_2_ content. For the 1% nano-ZrO_2_ content, the nano-ZrO_2_ particle tended to sink into the coating due to its high density during the curing process, and larger micro-channels emerged on the top of the coating in the initial stage, which led to the Z-1 coating having the lowest resistances (*R*_coat_ and *R*_ct_) and highest *Ceff*_coat_. When the nano-ZrO_2_ content increased to 3%, nano-ZrO_2_ particles were able to uniformly distribute in the coating and the hole sealing effect had an absolute advantage over agglomeration, which effectively improved the resistances (*R*_coat_ and *R*_ct_) and led to a decline in *Ceff*_coat_ in the Z-3 coating. As the nano-ZrO_2_ content reached 5%, the dominant status of the hole-sealing effect in the nano-particles was challenged by aggregation, which led to a decline in resistance (*R*_coat_ and *R*_ct_) and an increase in *Ceff*_coat_ of the Z-5 coating to some degree.

With a prolonged immersion time, there was a prominent decrease in the resistances (*R*_coat_ and *R*_ct_) and an increase in *Ceff*_coat_ for most coatings, due to the development of pathways. In particular, the *Ceff*_coat_ of the Z-1 coating decreased firstly and then increased with an increased immersion time, while the resistances (*R*_coat_ and *R*_ct_) increased and then decreased. This implies that 1% nano-ZrO_2_ takes effect when the corrosive medium permeates into the lower part of the Z-1 coating. Additionally, the hole-sealing effect of insoluble corrosion products [45] should not be neglected, as confirmed in the shift of the *R*_coat_ value.

After 192 h of immersion, the significant decrease in the resistance and increase in *Ceff*_coat_ indicated that the corrosion process had occurred. With an increase in the nano-ZrO_2_ content, the corrosion process was inhibited firstly and then promoted, and the inhibition effect of 3% nano-ZrO_2_ was the most significant. The *Ceff*_coat_, *R*_coat_ and *R*_ct_ of the Z-3 coating were 0.25, 31.90 and 9.86 times that of coating Z-0, respectively, which indicates a markedly enhanced water resistance, lower porosity and larger charge transfer resistance.

**Figure 11:**
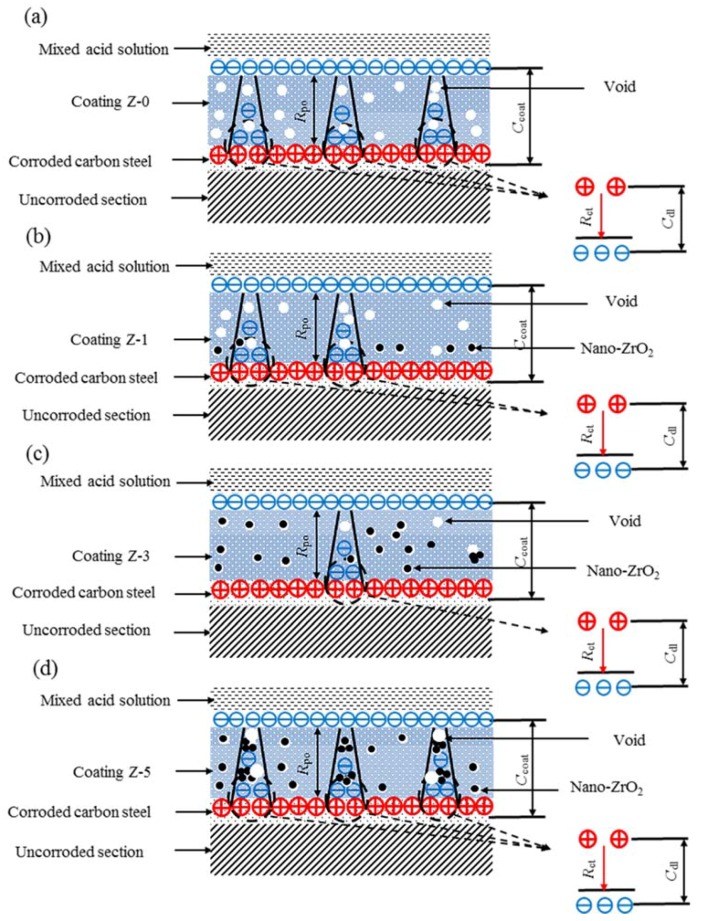
Schematic illustrations of corrosion process for the Z-0 (**a**), Z-1 (**b**), Z-3 (**c**) and Z-5 (**d**) coatings: 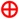: positive charge, 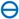: negative charge.

To further understand the anticorrosion mechanism of the nano-ZrO_2_-modified coatings in mixed acid solution, a schematic interpretation is depicted in Figure 11. As shown in Figure 11a, the Z-0 coating has abundant voids and defects, which provides preferential diffusion paths for corrosive species of Cl^−^, H^+^, and O_2_, etc. When a corrosive medium reaches the metal–coating interface, the following corrosion process for the substrate is proposed:

Anodic reaction:

Fe − 2e^−^ → Fe^2+^. (3)

Cathodic reaction:

2H^+^ + 2e^−^ → H_2_. (4)

The nano-ZrO_2_ addition did not change the reaction mechanism of the substrates. With an increase in the nano-ZrO_2_ content, the micro-pore channels gradually sealed, while excessive nano-ZrO_2_ caused agglomeration and produced larger micro-pore channels when corrosion occurred. The competition relationship between agglomeration and the hole-sealing effect of nano-particles was obviously visible. The addition of 1% nano-ZrO_2_ partially sealed micro-pore channels of the Z-1 coating (Figure 11b), and generated an incomplete nanostructure network, which was responsible for the limited improvement of corrosion resistance. In contrast, the addition of 3% nano-ZrO_2_ perfectly balanced the relationship between agglomeration and the sealing of micro-pore channels of nano-particles, and formed a relatively complete nanostructure network, which effectively improved the corrosion resistance of the Z-3 coating (Figure 11c). When the nano-ZrO_2_ content reached 5%, the corrosion resistance of the Z-5 coating dramatically reduced. Nano-particles still sealed micro-pore channels, while lots of agglomerations occurred for the Z-5 coating (Figure 11d). Thus, the Z-3 coating showed the best anticorrosion property.

## 4. Conclusions

The corrosion protection characterizations for nano-ZrO_2_-modified coatings were examined in mixed acid solution. Corrosion resistance was enhance for the coatings with 1% and 3% nano-ZrO_2_ contents, while it declined for the coating with 5% nano-ZrO_2_ content. The 3% nano-ZrO_2_ particle modified coating showed the best corrosion protection performance, as evidenced by EIS results. Visual assessments through SEM, AFM and elemental mapping observations were in accordance with the electrochemical results.

One percent nano-ZrO_2_, with an incomplete nanostructure network in the coating, showed a limited improvement to the anticorrosion property, and 5% nano-ZrO_2_ led to excessive aggregation and declined the adhesion of reinforced nanoparticles, which damaged the corrosion resistance. Three percent nano-ZrO_2_ possesses remarkable dispersion properties and a relatively complete nanostructure network in the coating, and was shown to balance the relationship between agglomeration and sealing of the micro-pore channels of nano-particles perfectly, which resulted in a minimum amount of diffusion of Cl, C, O and Fe, the lowest average roughness (5.94 nm), the highest C/O radio (4.55), and the best electrochemical properties (highest resistances and lowest *Ceff*_coat_).

## Figures and Tables

**Table 1:** Composition of coatings.

Sample Coding ^1^	Composition
Coating Z-0	65% phenolic-epoxy resin, 0.5% KH550 silane coupling agent, 34.1% 240 mesh glass flake, 0.4% BYK106 dispersant, 30% HN01 curing agent
Coating Z-1	Coating Z-0 + 1.49% nano-ZrO_2_ concentrate (1% nano-ZrO_2_)
Coating Z-3	Coating Z-0 + 4.48% nano-ZrO_2_ concentrate (3% nano-ZrO_2_)
Coating Z-5	Coating Z-0 + 7.46% nano-ZrO_2_ concentrate (5% nano-ZrO_2_)

^1^ Z represents nano-ZrO_2_. The number shows the wt % of ZrO_2_ nanoparticles embedded in the phenolic-epoxy coating.

**Table 2:** *R*_a_ and root mean square (*RMS*) values of different nano-ZrO_2_-modified coatings after 192 h of acid immersion, as deduced from AFM analysis.

Samples	*R*_a_ (nm)	*RMS* (nm)
Z-0 Coating	24.58	73.60
Z-1 Coating	12.79	27.44
Z-3 Coating	5.94	16.10
Z-5 Coating	20.42	52.03

**Table 3:** Atomic compositions (at %) derived from XPS survey spectra for the Z-0, Z-1, Z-3 and Z-5 coatings before and after acid immersion.

	Samples	Element	At %
Before immersion	Z-0, Z-1, Z-3 and Z-5 Coatings	C1s	79.86
		O1s	17.01
		N1s	1.8
After immersion	Z-0 Coating	C1s	77.82
		O1s	20.47
		N1s	1.71
	Z-1 Coating	C1s	80.37
		O1s	18.46
		N1s	1.16
	Z-3 Coating	C1s	80.77
		O1s	17.31
		N1s	1.93
	Z-5 Coating	C1s	76.99
		O1s	19.81
		N1s	3.21

**Table 4:** Fitting results of EIS for the Z-0, Z-1, Z-3, and Z-5 coatings after different acid immersion times.

Sample	Immersion Time/h	*R*_s_/Ω·cm^2^	*Q*_coat_/s^n^·Ω^−1^·cm^−2^	*α*_coat_	*Ceff*_coat_/F·cm^−2^	*R*_coat_/Ω·cm^−2^	*Q*_dl_/s^n^·Ω^−1^·cm^−2^	*α*_dl_	*R*_ct_/Ω·cm^2^	*Chsq*
Z-0 Coating	30 min	0.36	2.44 × 10^−9^	0.896	2.16 × 10^−10^	1.81 × 10^6^	2.02 × 10^−7^	0.2517	2.54 × 10^6^	1.30 × 10^−3^
	24 h	0.36	3.41 × 10^−10^	1	3.41 × 10^−10^	4.60 × 10^4^	1.67 × 10^−7^	0.5321	5.63 × 10^5^	2.21 × 10^−3^
	120 h	393	2.88 × 10^−9^	0.9113	7.59 × 10^−^^10^	1.48× 10^4^	1.99 × 10^−^^4^	0.1117	2.53 × 10^5^	3.59 × 10^−^^4^
	192 h	138	4.84 × 10^−7^	0.6298	1.66 × 10^−9^	3.26 × 10^3^	5.58 × 10^−4^	0.4868	2.84 × 10^4^	1.94 × 10^−4^
Z-1 Coating	30 min	0.36	2.10 × 10^−9^	0.9337	4.73 × 10^−10^	9.51 × 10^4^	1.17 × 10^−6^	0.296	3.70 × 10^5^	2.12 × 10^−3^
	24 h	0.37	3.88 × 10^−10^	1	3.88 × 10^−10^	1.11 × 10^5^	4.76 × 10^−7^	0.3895	6.08 × 10^5^	1.16 × 10^−3^
	120 h	0.36	7.46 × 10^−9^	0.8793	4.97 × 10^−10^	6.89 × 10^4^	2.24 × 10^−6^	0.1965	3.58 × 10^5^	9.63 × 10^−4^
	192 h	723	4.89 × 10^−8^	0.7395	1.32 × 10^−9^	5.10 × 10^4^	2.47 × 10^−5^	0.4372	1.52 × 10^5^	3.34 × 10^−3^
Z-3 Coating	30 min	0.36	2.16 × 10^−9^	0.8403	4.02 × 10^−11^	8.26 × 10^7^	1.07 × 10^−7^	0.4962	1.11 × 10^8^	1.68 × 10^−3^
	24 h	0.36	1.09 × 10^−9^	0.9344	2.37 × 10^−10^	2.38 × 10^6^	1.76 × 10^−8^	0.3601	8.67 × 10^6^	2.37 × 10^−3^
	120 h	0.36	1.52 × 10^−9^	0.9331	3.28 × 10^−10^	1.26 × 10^6^	3.28 × 10^−7^	0.4515	1.31 × 10^6^	2.15 × 10^−3^
	192 h	0.36	1.11 × 10^−9^	0.9572	4.21 × 10^−10^	1.04 × 10^5^	8.61 × 10^−6^	0.2803	2.80 × 10^5^	3.97 × 10^−4^
Z-5 Coating	30 min	0.36	2.47 × 10^−9^	0.8815	1.50 × 10^−10^	3.21 × 10^6^	2.67 × 10^−8^	0.2855	4.09 × 10^7^	2.55 × 10^−3^
	24 h	0.36	2.34 × 10^−9^	0.9338	5.31 × 10^−10^	2.01 × 10^5^	2.61 × 10^−7^	0.3371	1.49 × 10^6^	2.29 × 10^−3^
	120 h	228	1.19 × 10^−^^7^	0.6911	1.08 × 10^−9^	4.31 × 10^4^	4.41 × 10^−^^5^	0.3982	4.53 × 10^4^	4.51 × 10^−^^4^
	192 h	67.3	2.35 × 10^−8^	0.8619	2.74 × 10^−9^	1.41× 10^3^	5.10 × 10^−4^	0.2736	5.76 × 10^3^	4.13 × 10^−4^
